# Total Flavonoids from* Carya cathayensis* Sarg. Leaves Alleviate H9c2 Cells Hypoxia/Reoxygenation Injury via Effects on miR-21 Expression, PTEN/Akt, and the Bcl-2/Bax Pathway

**DOI:** 10.1155/2018/8617314

**Published:** 2018-12-05

**Authors:** Ruibin Jiang, Yan Guo, Nipi Chen, Chengxian Gao, Zhishan Ding, Bo Jin

**Affiliations:** ^1^College of Life Science, Zhejiang Chinese Medical University, Hangzhou 310053, China; ^2^Cancer Research Institute, Zhejiang Cancer Hospital, Hangzhou 310022, China; ^3^College of Medical Technology, Zhejiang Chinese Medical University, Hangzhou 310053, China

## Abstract

This study aimed to investigate whether the total flavonoids (TFs) from* Carya cathayensis* Sarg. leaves alleviate hypoxia/reoxygenation (H/R) injury in H9c2 cardiomyocytes and to explore potential mechanisms. H9c2 cells pretreated with TFs for 24h were exposed to H/R treatment. The results indicated that TFs significantly alleviate H/R injury, which include inhibiting apoptosis and enhancing antioxidant capacity. The protective effects of TFs resulted in higher expression of miR-21 in H/R-induced H9c2 cells than that of controls, which in turn upregulated Akt signaling activity via suppressing the expression of PTEN together with decreasing the ratio of Bax/Bcl-2, caspase3, and cleaved-caspase3 expression in H/R-induced H9c2 cells. Conversely, blocking miR-21 expression with miR-21 inhibitor effectively suppressed the protective effects of TFs against H/R-induced injury. Our study suggests that TFs can decrease cell apoptosis, which may be mediated by altering the expression of miR-21, PTEN/Akt, and Bcl/Bax.

## 1. Introduction

Cardiac ischemia/reperfusion (I/R) injury is a serious disease and threatens human health [1]. Reperfusion treatment has a potential risk of worsening tissue damage after ischemia, which can accelerate the deterioration of cardiac function [2]. The myocardial apoptosis and inflammation have been recognized as features of I/R injury. In order to fully understand the mechanisms of I/R injury and to find novel therapeutic strategies, further research is stilled urgently needed [3].

microRNAs (miRNAs) are important regulators of target messenger RNA translation by binding mainly to complementary sequences of the 3′ untranslated region of target messenger RNA transcripts thereby leading to RNA degradation and/or inhibition of protein synthesis [4]. miRNAs have been implicated as transcriptional regulators in a wide range of biological processes determining cell fate, stress response, proliferation, or death [5]. A multitude of studies has demonstrated the role of miRNAs in chronic cardiovascular or renal disease processes [6–11]. miR-21 was found to be highly deregulated in mouse cardiac tissue after cardiac ischemic preconditioning [12]. In murine cardiomyocytes miR-21 was found to protect from hypoxia/reoxygenation (H/R)-induced cell apoptosis via regulation of its target gene PDCD4 [13]. Despite the recent surge about miRNA discoveries for cardiac I/R injury, there is still very little known about the mechanism details because of the complexity of cellular events and the interference of other risk factors.

*Carya cathayensis* Sarg., known as Chinese hickory, is a native species to China according to the “Compendium of Materia Medica” [14].* Carya cathayensis* Sarg. has been used to keep fit and prevent cardiovascular diseases for a long time in the folk [15]. Numerous studies have shown that flavonoids can prevent cardiovascular disease [16–19]. We recently have isolated flavonoids from the leaves of* Carya cathayensis* Sarg., which contained five main components [20]. Cardamonin, Pinostrobin, Wogonin, and Chrysin had antioxidant activities [21–23]. We found that cardamonin could regulate miR-21 expression [24]. Wogonin could suppress apoptosis in rats experienced myocardial I/R [25]. Chrysin regulated miR-18a, miR-21, and miR-221 genes in gastric cancer cell line [26]. Moreover, we have found that TFs can increase the activity of superoxide dismutase (SOD) and reduce the level of malonaldehyde (MDA) together with lactate dehydrogenase (LDH) in H9c2 cells. We also demonstrated that TFs alleviate H/R injury and increase the expression of miR-21. We hypothesized that TFs suppresses H/R injury by regulating miR-21. To test this hypothesis, we attempted to investigate whether TFs can exert its cardioprotective effect and the effects of TFs on miR-21 in H9c2 cells.

## 2. Methods and Methods

### 2.1. Materials and Reagents

The total flavonoids (TFs) were extracted from the leaves of* Carya cathayensis* Sarg. with 40% ethanol and enriched total flavonoids by polyamide [27]. The TFs were grounded into a powder (10.0mg) and added with 10.0ml of absolute ethanol. It sonicated to completely dissolve it and used the membrane filtration. The content of TFs (wogonin, chrysin, cardamom, pinostrobin chalcone, and pinocembrin) was determined by UltiMate 3000 high performance liquid chromatography. The column temperature was 30°C. The mobile phase was 0.1% aqueous acetic acid-methanol (40:60), the flow rate was 1.0ml/min, the detection wavelength was 262nm, 272nm, 284nm, and 420nm, and the injection amount was 10*μ*L. The leaves collected form Tianmu Mountain district, a cross area of Zhejiang and Anhui provinces in China [28], in this experiment were identified by professor Zhishan Ding of Zhejiang Chinese Medical University. A voucher specimen of the plant material used in this study has been deposited in molecular biology laboratory of Zhejiang Chinese Medical University (NO.LCC-20160915-G).

### 2.2. Cell Culture

The H9c2 cells were purchased from the Cell Bank of Chinese Academy of Science (Shanghai, China). The cells were cultured in Dulbecco's modified Eagle's medium supplemented with 10% fetal bovine serum. Cells were maintained in a humidified incubator consisting of 5% CO_2_ and 95% air at 37°C.

### 2.3. H/R Model and Drug Treatment

Cells were exposed to 10mM Na_2_S_2_O_4_ for 7h in culture medium deprived of serum. After hypoxia, the cells were reoxygenated under normoxic conditions (reoxygenation) for 12h in normal medium before they were used for further analysis. To investigate the effect of TFs on H/R injury, H9c2 were pretreated with TFs at different concentrations (2.5*μ*g/ml, 5*μ*g/ml, or 10*μ*g/ml) for 24h.

### 2.4. Cell Viability Assay

The MTS assay was used to investigate the anti-H/R effects of TFs on H9c2 cells through adopting the CellTiter 96® AQueous One Solution Cell Proliferation Assay (Promega, USA). Briefly, 5x10^3^ cells were seeded into 96-well plates overnight. After treatment, 20*μ*l MTS solution was added to each well and incubated at 37°C for additional 2h. Finally, absorbance of the samples was measured at 490nm using a microplate reader (BioTek instrument, America).

### 2.5. miRNA Transfection

Cells in the exponential phase of growth were plated in six-well plates at 2×10^5^ cells/plate and cultured for 24h. Then, the cells were transfected with the miR-21 mimic (50nM), miR-21 inhibitor (100nM), or control miRNA using Lipofectamine RNAiMAX (Invitrogen) according to the manufacturer's protocols. After 24h incubation, cells were further cultured in medium containing TFs for 24h before being subjected to H/R treatment.

### 2.6. Measurement of Cellular LDH, MDA Level, and SOD Activity

Levels of LDH, MDA, and the activity of SOD were measured in the cell culture medium using assay kits (Jiancheng Bioengineering Institute, China).

### 2.7. Detection of Apoptotic Cells with Flow Cytometry

The work ascertained the programmed death of H9c2 cells triggered by H/R through adopting an Annexin V-FITC/PI kit (Jiancheng Bioengineering Institute, China) abiding by the flow cytometry. Briefly, the cells were washed adopting PBS chilled, and subsequently trypsin was given to the cells, which were resuspended in 50*μ*L binding buffer. The cells were labeled with 10*μ*L PI and 5*μ*L Annexin V-FITC solution at the normal temperature for 10min in the dark. Flow cytometry (Millipore, America) was used to examine the fluorescent signals.

### 2.8. Hoechst-PI Staining

Hoechst-PI staining was used to detect H9c2 apoptosis rate. After treatment, Hoechst 33342 (5mg/ml) and PI (5mg/ml) were added for 10min at room temperature in the dark. Cells were visualized and scored using a phase-contrast and fluorescence microscope (Nikon, Japan). The results were analyzed using the Image J software.

### 2.9. Isolation of RNA and Real-Time RT-PCR

Total RNA was extracted with TRIzol reagent (Invitrogen), and cDNA was generated using a commercial kit (Invitrogen) with PCR conditions of 37°C for 60min, and 85°C for 5s, and then stored at 4°C. Real-time PCR was performed with SYBR® Premix EX Taq TM II (Tli RNaseH plus) (Takara, China) with PCR conditions of denaturation at 94°C for 1min, and then 94°C for 30s, 55°C for 30s, and 72°C for 30s for 40 cycles. The primer sequences are as follows: miR-21: ACGTTGTGTAGCTTATCAGTG.

### 2.10. Western Blot Analysis

Proteins from cell lysates or tissue lysates were separated by a 10% SDS polyacrylamide gel electrophoresis and transferred to a polyvinylidene fluoride membrane. After being blocked in 5% nonfat milk, protein blots were probed with a primary antibody followed by incubation with a peroxidase-conjugated secondary antibody. The primary antibodies included HIF-1*α* (1:1000; ImmunoWay), Bax (1:1000; ImmunoWay), Bcl-2 (1:1000; ImmunoWay), caspase3 (1:1000; ImmunoWay), cleaved-caspase3 (1:1000; ImmunoWay), and PTEN (1:500; ImmunoWay). Chemiluminescence was detected by the ECL-plus kit (Beyotime). Band intensity was quantified by Image J software.

### 2.11. Statistical Analysis

All values are reported as the mean ± SEM. Statistical analysis was performed using one-way ANOVA and comparisons between two groups were performed using least significant difference (LSD) test. P<0.05 was considered to indicate statistical significance.

## 3. Results

### 3.1. TFs Was Standardized by HPLC

**Figure 1:**
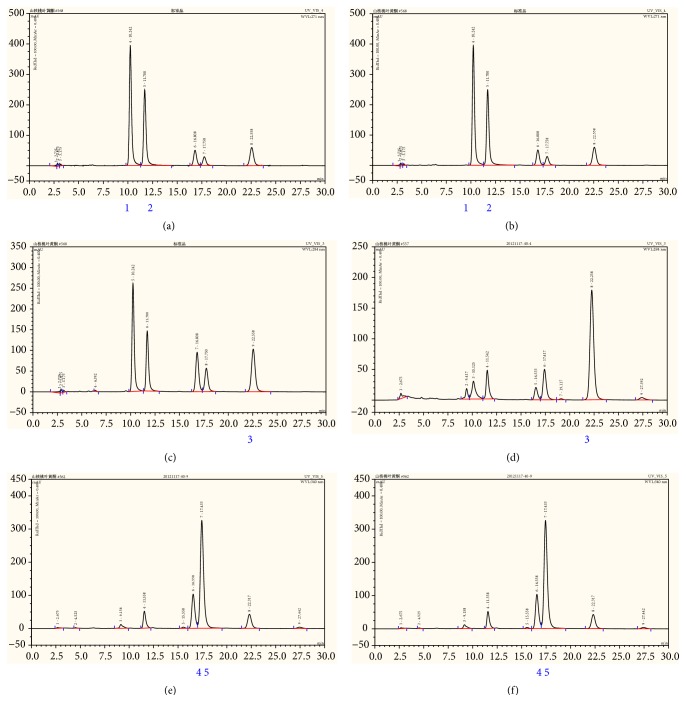
TFs was standardized by HPLC. (a-b) Analysis of TFs by HPLC at 271nm. (a) Standard wogonin and cardamom, (b) TFs. (c-d) Analysis of TFs by HPLC at 284nm. (c) Standard chrysin, (d) TFs. (e-f) Analysis of TFs by HPLC at 342nm. (e) Standard pinostrobin chalcone and pinocembrin, (f) TFs. 1: Wogonin; 2: Cardamom; 3: Chrysin; 4: Pinostrobin chalcone; and 5: Pinocembrin.

As shown in Figures 1(a)–1(f), among the TFs, the content of five known substances (wogonin, chrysin, cardamom, pinostrobin chalcone, and pinocembrin) was 58.6%.

### 3.2. TFs Reversed the Viability of H9c2 Cells

**Figure 2:**
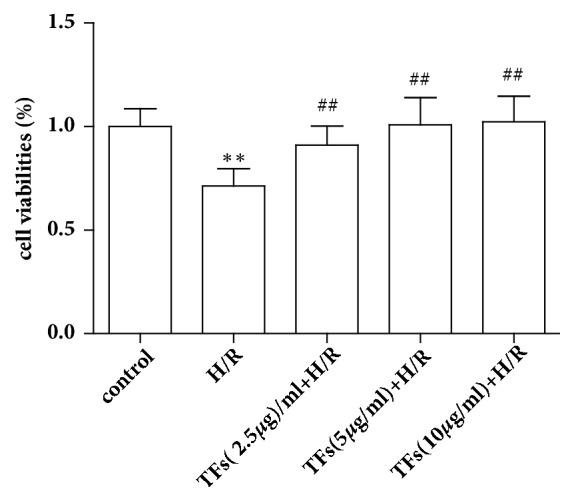
TFs reversed the viability of H9c2 cells. Cell viability was measured with the MTS assay. All values are expressed as mean±SEM (n=3). ^*∗*^p<0.01 and ^*∗∗*^p<0.01 vs. control group, ^#^p<0.05 and ^##^p<0.01 vs. H/R group.

H9c2 cells were exposed to 10mM Na_2_S_2_O_4_ for 7h followed by reoxygenation for another 12h, and the results revealed that H/R injury resulted in decreasing cells viability. However, TFs pretreatment reversed the viability of H9c2 cells after H/R injury (Figure 2).

### 3.3. TFs Affected the Level of LDH

**Figure 3:**
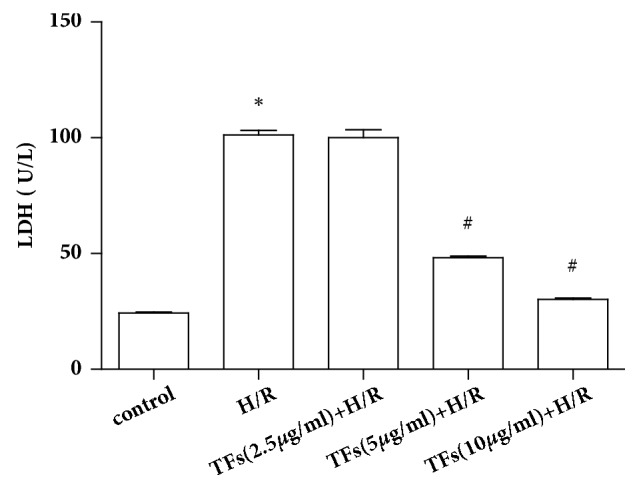
TFs affected the level of LDH. Cell injury was determined by measuring LDH release in H/R-injured H9C2 cells. All values are expressed as mean±SEM (n=3). ^*∗*^p<0.01 and ^*∗∗*^p<0.01 vs. control group, ^#^p<0.05 and ^##^p<0.01 vs. H/R group.

The level of LDH in medium was considered as myocardial injury marker enzymes, as shown in Figure 3. The results of LDH assay indicated that H/R injury increased LDH significantly (P<0.05). After incubation with TFs (5,10ug/ml) to H/R injury, LDH releases decreased respectively in H9c2 cells (P<0.05).

### 3.4. TFs Affected Antioxidant Systems and Lipid Peroxidation

**Figure 4:**
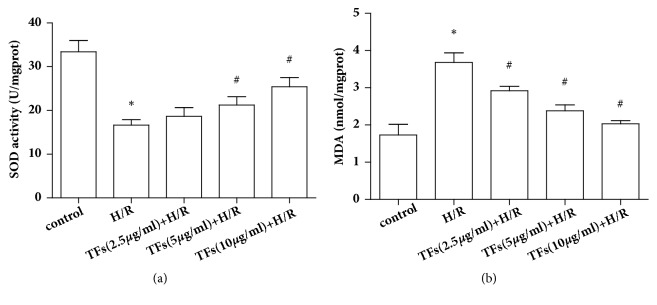
TFs affected antioxidant systems and lipid peroxidation. (a) SOD activity was measured by a commercial SOD kit. (b) The level of MDA was evaluated by a MDA assay kit. All values are expressed as mean±SEM (n=3). ^*∗*^p<0.01 and ^*∗∗*^p<0.01 vs. control group, ^#^p<0.05 and ^##^p<0.01 vs. H/R group.

Oxidative stress caused by reperfusion can lead to lipid peroxidation. The results of lipid peroxidation product MDA and antioxidant system of SOD were shown in Figures 4(a) and 4(b). As shown in Figure 4(a), the activity of SOD significantly decreased in H/R-injured H9c2 cells compared with that in the control group (P<0.05). When H/R-injured H9c2 cells were incubated with TFs, SOD activity was significantly increased compared with the H/R group (P<0.05). In contrast, as shown in Figure 4(b), TFs pretreatment efficiently suppressed H/R-induced MDA production in H9c2 cells (P<0.05).

### 3.5. TFs Prevented H9c2 Cells from Apoptosis

**Figure 5:**
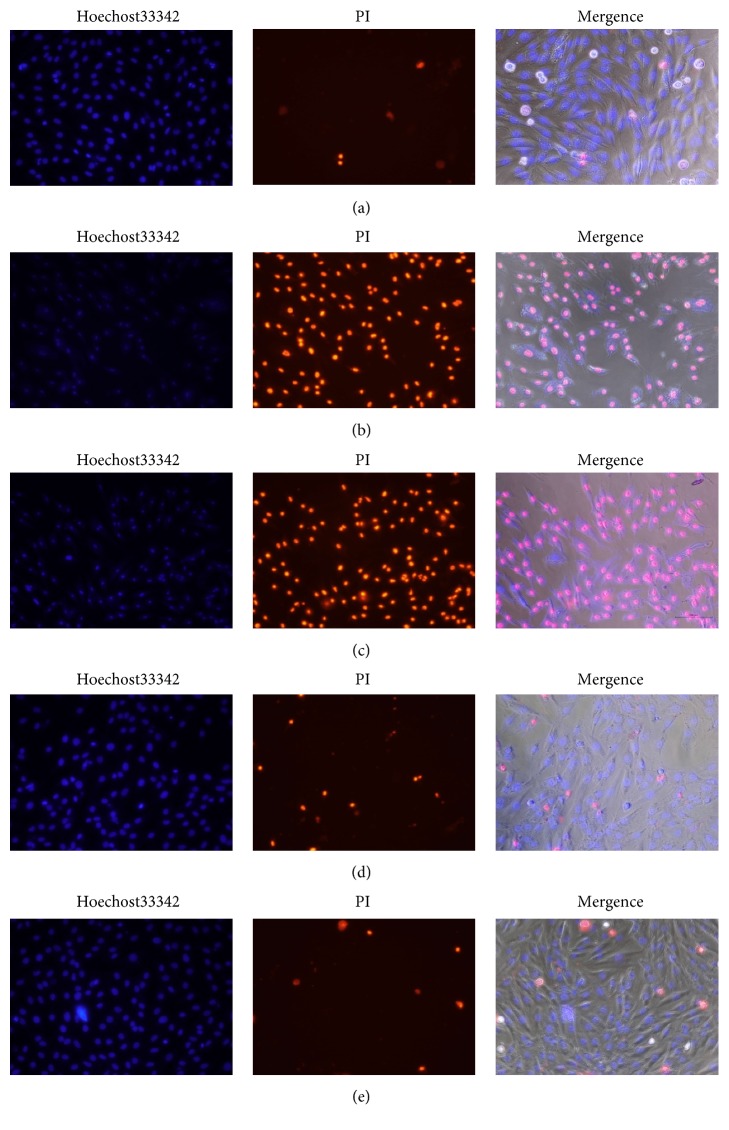
TFs prevented H9c2 cells from apoptosis. Representative images of Hoechst 33342 and PI staining. (a) is control group; (b) is H/R group; (c) is H/R+TFs (2.5ug/ml) group; (d) is H/R+TFs (5ug/ml) group; and (e) is H/R+TFs (10ug/ml) group.

As illustrated in the microphotographs of Figure 5, the nuclei of the dead cells were penetrated by PI, which released red fluorescence, while the living cells were only stained with Hoechst 33342 and thus exhibited blue fluorescence. After H/R injury, the number of apoptotic cells (red fluorescence) significantly increased. In contrast, pretreatment with TFs (2.5-10ug/ml) in the presence of 10mM Na_2_S_2_O_4_ for 7h elevated cell viability.

### 3.6. TFs Affected the Expression of Apoptosis-Related Protein

**Figure 6:**
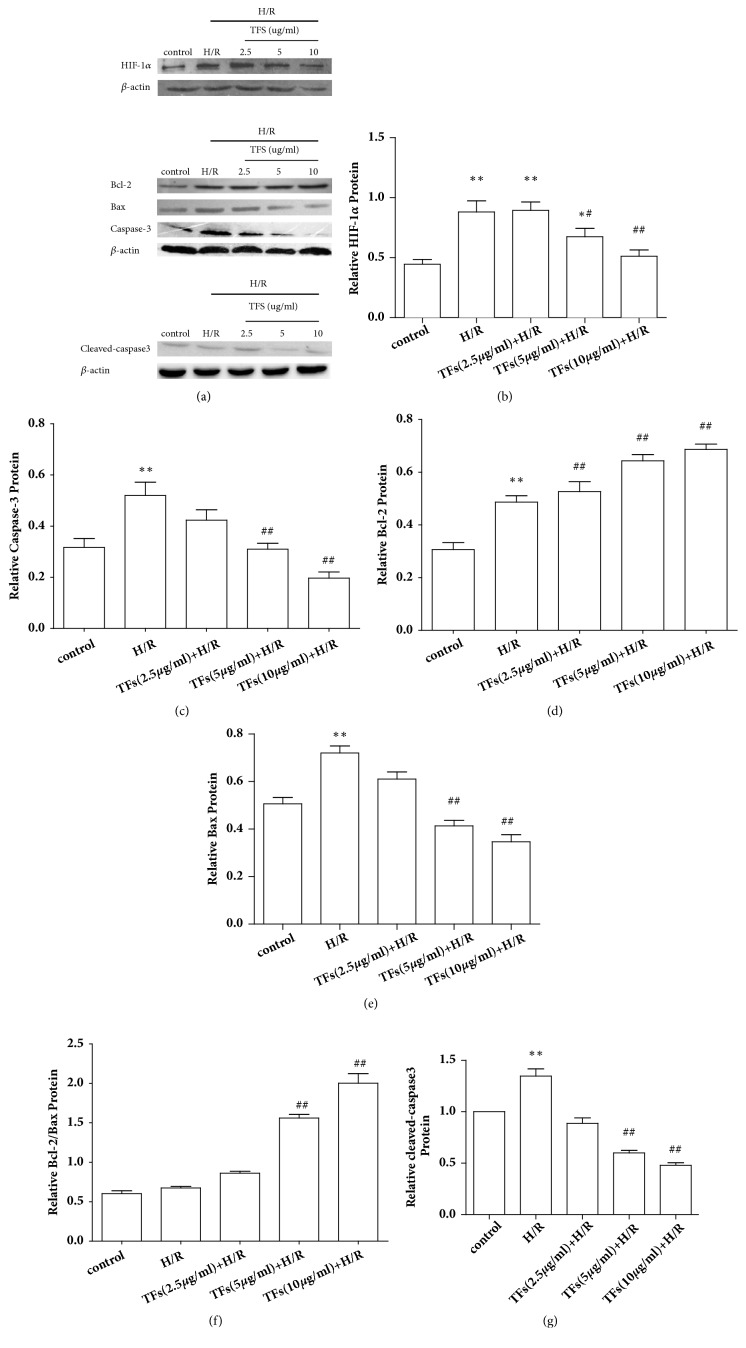
TFs increased the Bcl-2 protein expression but decreased the HIF-1*α*, Bax, and caspase-3 protein expression in H9c2 cells. (a-g) Representative images of WB analysis and the semiquantification of HIF-1*α*, Bcl-2, Bax, caspase3, and cleaved-caspase3 protein expression in H9c2 cells were shown. All values are expressed as mean±SEM (n=3). ^*∗*^p<0.01 and ^*∗∗*^p<0.01 vs. control group, ^#^p<0.05 and ^##^p<0.01 vs. H/R group.

To further demonstrate the antiapoptotic effect of TFs, the levels of apoptosis-related proteins, such as HIF-1*α*, Bcl-2, Bax, caspase3, and cleaved-caspase3 expression in H9c2 cells, were determined by Western blotting. TFs reduced the expressions of proapoptotic protein including HIF-1*α*, Bax, caspase3, and cleaved-caspase3 (P<0.01), while upregulated antiapoptotic protein Bcl-2 (P<0.01) with an optimal concentration of 10*μ*g/ml rather than a dose of 2.5ug/ml (Figures 6(a)–6(g)).

### 3.7. TFs Activated miR-21 Expression

**Figure 7:**
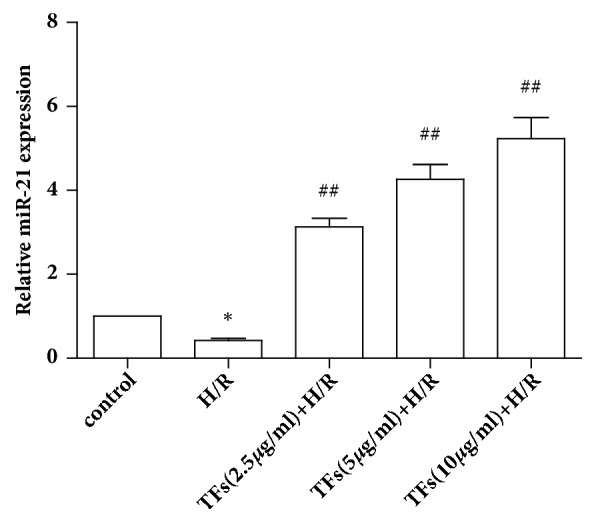
TFs increased the miR-21 expression. Quantitative analysis of miR-21 in H9c2 cells was shown. All values are expressed as mean±SEM (n=3). ^*∗*^p<0.01 and ^*∗∗*^p<0.01 vs. control group, ^#^p<0.05 and ^##^p<0.01 vs. H/R group.

To investigate the potential effects of TFs on H/R injury, miR-21 expression was detected using qPCR. As shown in Figure 7, H/R injury markedly suppressed miR-21 expression in H9c2 cells, as compared with in the control group (P<0.05). Conversely, TFs significantly upregulated miR-21 expression after H/R injury (P<0.01).

### 3.8. TFs Retarded the Apoptosis Rate of H9c2 Cells

**Figure 8:**
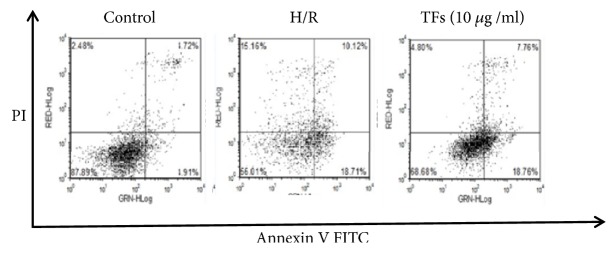
TFs alleviated H/R injury in H9c2 cells. The cell apoptosis rate was detected with an Annexin V-FITC/PI kit. The values are expressed as the mean±SEM (n=3). ^*∗*^p<0.05 and ^*∗∗*^p<0.01 vs. control group, ^#^p<0.05 and ^##^p<0.01 vs. H/R group.

Figure 8 showed that annexin+/PI and annexin+/PI+ were substantially decreased in the TFs-treated cells. This result suggested that the apoptosis rate of H9c2 cells was significantly increased by H/R challenge, and the cell apoptosis rate increased to 15.16% of that of the control group. However, these changes were markedly reversed by TFs preincubation. These results suggested that TFs is capable of rescuing H9c2 cells from H/R-induced apoptotic death.

### 3.9. miRNA-21 Expression Affected the Protective Effect of TFs

**Figure 9:**
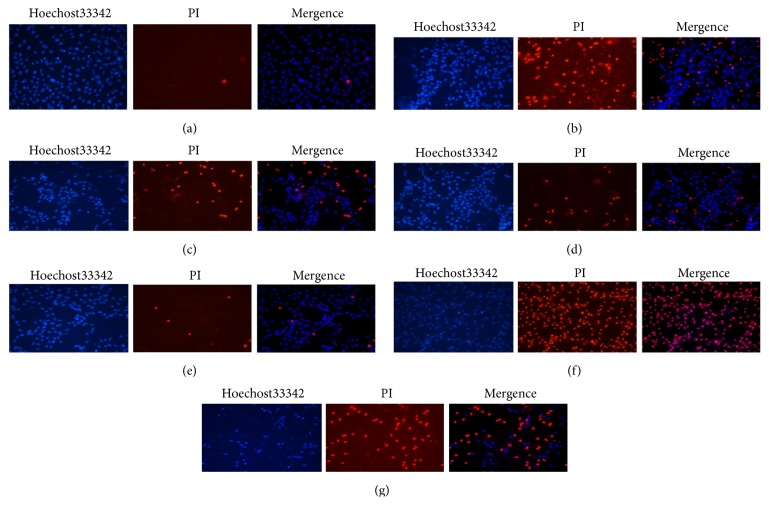
miR-21 expression affected the protective effect of TFs. Representative images of Hoechst 33324 and PI staining were shown. (a) was control group; (b) was H/R group; (c) was H/R+TFs (10ug/ml) group; (d) was H/R+mimic group; (e) was H/R+miR-21 mimic+TFs (10ug/ml) group; (f) was H/R+inhibitor group; and (g) was H/R+miR-21 inhibitor+TFs (10ug/ml) group.

When H9c2 cells were transfected with miR-21 mimic and inhibitor, the number of apoptotic cells significantly decreased or increased. With TFs and miR-21 mimic treatment, the number of apoptotic cells significantly decreased more obviously compared to cells treated only with TFs (Figure 9).

### 3.10. miR-21 Expression Affected PTEN/Akt Protein Expression

**Figure 10:**
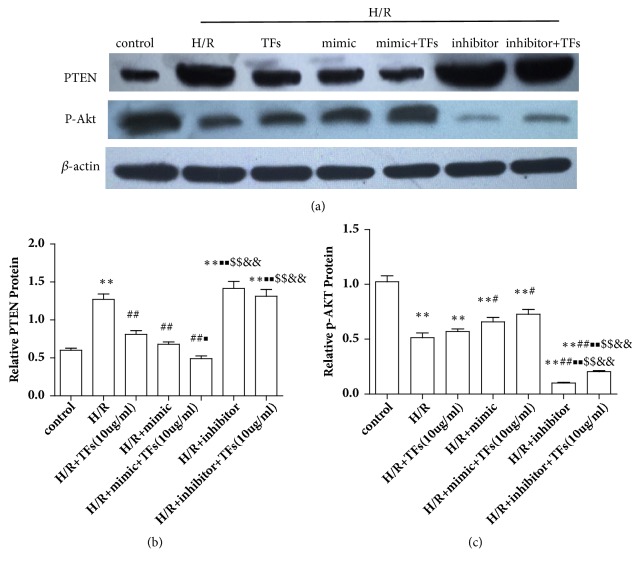
miR-21 expression affected the PTEN/Akt protein expression. (a-c) Representative images of WB analysis and the semiquantification of PTEN/p-Akt expression in H9c2 cells were shown. All values are expressed as the mean±SEM (n=3). ^*∗*^p<0.01 and ^*∗∗*^p<0.01 vs. control group, ^#^p<0.05 and ^##^p<0.01 vs. H/R group. ^■^p<0.01 and ^■■^p<0.01 vs. H/R+TFs(10ug/ml) group, ^$^p<0.01 and ^$$^p<0.01 vs. H/R+miR-21 mimic group, and ^&^p<0.01 and ^&&^p<0.01 vs. H/R+miR-21 mimic+TFs(10ug/ml) group.

PTEN was traditionally known to generate effects via suppression of p-Akt. To further explore the mechanism underlying TFs-induced miR-21-mediated cardiac protection in vitro, the effects of miR-21 expression promote or inhibit on PTEN/p-Akt protein expression were detected. The protein expression levels of p-Akt in H/R-induced H9c2 cells were markedly elevated following treatment with TFs compared with the control group (P<0.01). Notably, blocking miR-21 expression significantly increased PTEN protein expression in H9c2 cells (P<0.01), thereby inhibiting the expression of p-Akt (Figures 10(a)–10(c)).

### 3.11. miR-21 Expression Affected Bcl-2/Bax and Caspase3 Protein Expression

**Figure 11:**
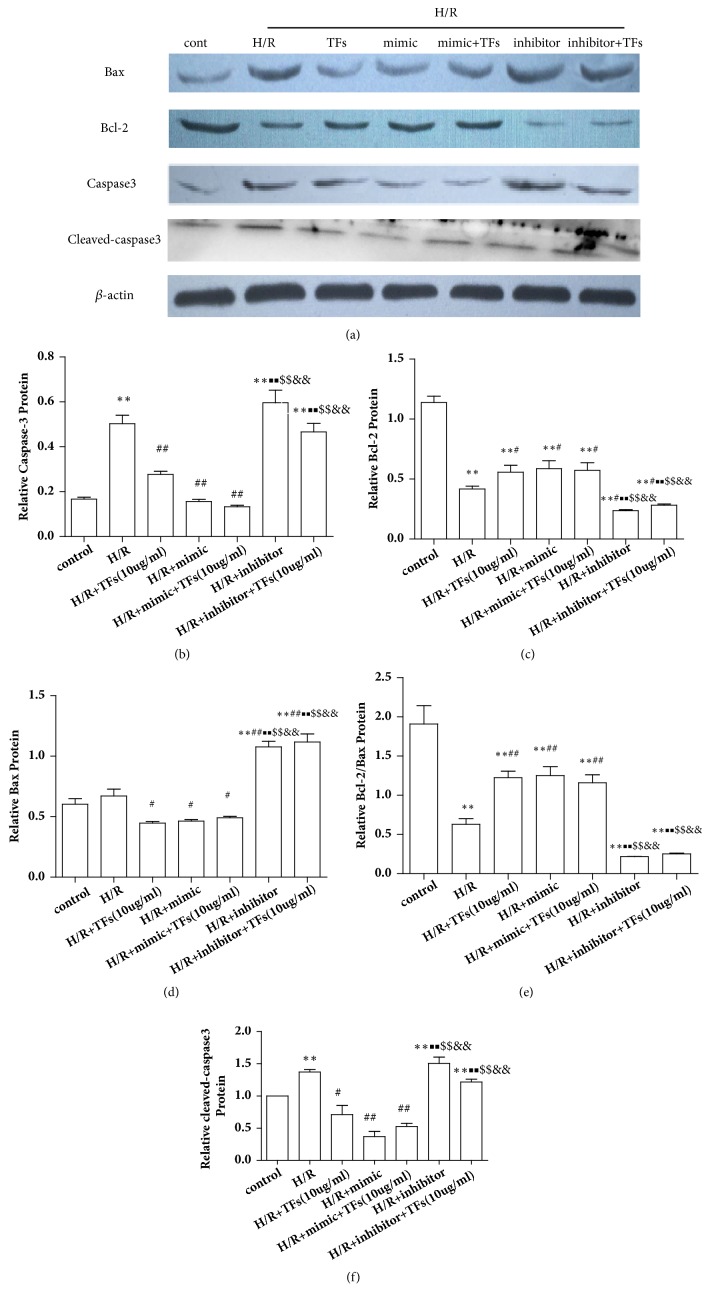
miR-21 expression affected Bcl-2/Bax, caspase3, and cleaved-caspase3 protein expression. (a-f) Representative images of WB analysis and the semiquantification of Bcl-2/Bax, caspase3, and cleaved-caspase3 expression in H9c2 cells were shown. All values are expressed as the mean±SEM (n=3). ^*∗*^p<0.01 and ^*∗∗*^p<0.01 vs. control group, ^#^p<0.05 and ^##^p<0.01 vs. H/R group. ^■^p<0.01 and ^■■^p<0.01 vs. H/R+TFs (10ug/ml) group, ^$^p<0.01 and ^$$^p<0.01 vs. H/R+miR-21 mimic group, and ^&^p<0.01 and ^&&^p<0.01 vs. H/R+miR-21 mimic+TFs (10ug/ml) group.

The effects of cardiac miR-21 expression promote or inhibit on Bcl-2/Bax and caspase3 protein expression were detected. As shown in Figures 11(a)–11(f), treatment with TFs markedly increased Bcl-2 protein expression in H/R-induced H9c2 cells compared with the control group (P<0.01). However, blocking miRNA-21 expression significantly inhibited Bcl-2 protein expression in H/R-induced H9c2 cells (P<0.01). Furthermore, treatment with TFs markedly reduced Bax, caspase3, and cleaved-caspase3 protein expression in H/R-induced H9c2 cells, compared with the control group (P<0.01). Conversely, blocking miR-21 expression significantly augmented Bax, caspase3, and cleaved-caspase3 protein expression in H/R-induced H9c2 cells (P<0.01).

## 4. Discussion

Cardiac I/R injury referred to a series of myocardial episodes, which were caused by coronary recanalization and myocardial reperfusion after myocardial ischemia. I/R injury induced complex physiological and pathological alterations [29]. Following myocardial ischemia, cardiac cells exhibit an energy supply reduction, cell membrane permeability increase, dysfunction of the membrane pump, and LDH leakage [30]. The increased degree of enzymatic activity in the serum can reflect the extent of myocardial damage [31]. MDA is the end production after reactive oxygen species attack unsaturated fat in cell membrane system, thus its content reflects lipid peroxidation in cells. SOD is an antioxidant enzyme which can catalyze reduction of the superoxide (O_2_^−^) radical into either molecular oxygen (O_2_) or hydrogen peroxide [32]. The results of the present study indicated that the potential protective effects of TFs effectively reduced these alterations in H9c2 cells following H/R injury.

miRNA can regulate target RNA either by repression or by promotion [33]. Recent studies have revealed that the levels of miRNA expressed in myocardia are involved in regulation of heart development, cell apoptosis, angiogenesis, hypertension, and myocardial infarction, as well as other cardiac physiological functions; therefore, appropriately regulating expression of these miRNA can reduce and even reverse the pathological process [4, 6, 34]. Furthermore, myocardial ischemia may induce damage to myocardial cells via miRNA expression [35]. It is of great significance to further study the regulatory mechanisms of miRNA in cardiac I/R injury. In the current study, the protective effects of TFs significantly promoted the expression of miR-21 in H9c2 cells following H/R injury. We found that miR-21 levels were higher in H9c2 pretreated with TFs compared with the H/R group. These findings promoted us to hypothesize that miR-21 levels can be regulated by TFs, and miR-21 may be the target gene of TFs. We then transfected miR-21 mimic and miR-21 inhibitor in H9c2 cells to determine the functional consequence of altered miR-21 expression in H/R. Previous studies have shown that miR-21 repressed the target gene level.

PTEN is a downstream gene of miR-21 and plays an important role in cell apoptosis [36]. PTEN/Akt signaling pathway is a major regulatory pathway of cell apoptosis, and PTEN can promote cell apoptosis by inhibiting Akt phosphorylation; Akt, in turn, can inhibit cell apoptosis by regulating Bcl family and caspase family [37]. Bcl-2 exerts an inhibitory function on apoptosis. Bcl-2 and Bax proteins are the two main members of the Bcl-2 multigene family [38]. Bcl-2 inhibits apoptosis, whereas Bax exerts a proapoptotic effect. In particular, caspase3 is the central molecule in apoptosis, and its activation is regulated by a series of signal transduction cascades, among which the interaction between antiapoptotic Bcl-2 and proapoptotic Bax proteins plays a vital role. The present study demonstrated that TFs significantly augmented the Bcl-2/Bax ratio in H9c2 cells following H/R injury. To further analyze the mechanism underlying TFs-induced miR-21-mediated cardiac protection in vitro, the effects of cardiac miR-21 expression on PTEN/Akt and Bcl-2/Bax protein expression were detected. In our study, the levels of PTEN decreased when H9c2 cells transfected with miR-21 mimic, and otherwise increased when H9c2 cells transfected with miR-21 inhibitor. We found that the levels of PTEN, caspase3, and cleaved-caspase3 protein decreased when miR-21 was overexpressed, while the levels of Bcl-2/Bax and p-Akt protein increased. Using TFs and miR-21 mimic together further reduced the expression of PTEN, caspase3, and cleaved-caspase3 protein and further increased the expression of Bcl-2/Bax and p-Akt protein, all of which led to a reduction in cell apoptosis. The levels of PTEN, caspase3, and cleaved-caspase3 protein increased when the expression of miR-21 was inhibited, while the levels of Bcl-2/Bax decreased and the expression of p-Akt protein was inhibited. The results confirmed that blocking miR-21 expression reversed the protective effects of TFs against H/R injury and influenced the expression of the PTEN/Akt and Bcl-2/Bax pathway.

## 5. Conclusions

**Figure 12:**
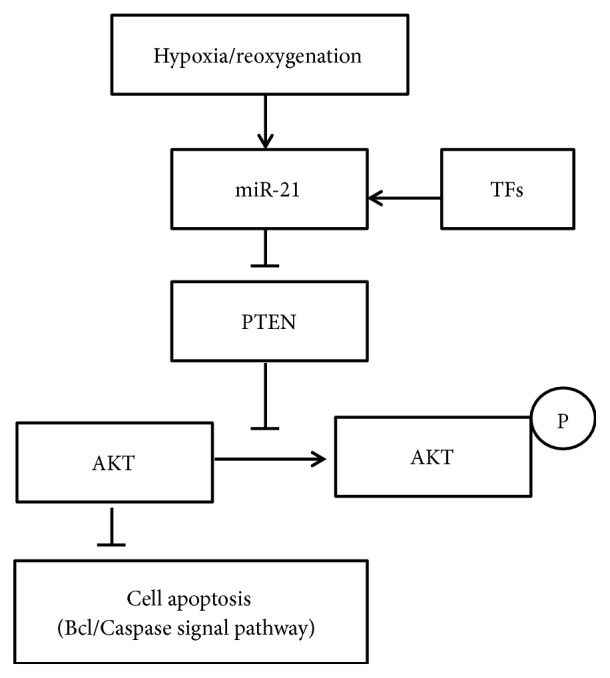
Schematic presentation of mechanism for TFs-induced inhibition of apoptosis in H/R-treated H9c2 cells through miR-21-mediated pathways.

The present study demonstrates that TFs alleviate H/R injury in HUVEC, possibly by inhibiting apoptosis, and the protection required the activation of miR-21 expression and PTEN/Akt pathway (Figure 12). The data of the present study suggest that TFs may be a potential therapy drug for the treatment of cardiac I/R injury. But we have only verified the preventive effect of TFs on I/R injury and confirmed the effect of miR-21 on TFs in preventing I/R injury with the expression of apoptosis-related protein. We do not explore the relationship between miR-21 and the PTEN/Akt or Bcl-2/Bax pathway, which we will study in the future.

## Data Availability

The data used to support the findings of this study are available from the corresponding author upon request.
